# Quantitative measurement of finger usage in stroke hemiplegia using ring-shaped wearable devices

**DOI:** 10.1186/s12984-023-01199-4

**Published:** 2023-06-06

**Authors:** Naoya Yamamoto, Takato Matsumoto, Tamami Sudo, Megumi Miyashita, Toshiyuki Kondo

**Affiliations:** 1 Department of Computer and Information Sciences, Graduate School of Engineering, Tokyo University of Agriculture and Technology, 2-24-16, Naka-cho, Koganei, Tokyo Japan; 2 Department of Rehabilitation, Shonan Keiiku Hospital, 4360, Endo, Fujisawa, Kanagawa Japan

**Keywords:** Physical activity, Activities of daily living, Finger, Upper limb, Rehabilitation, Stroke

## Abstract

**Background:**

In post-stroke rehabilitation, positive use of affected limbs in daily life is important to improve affected upper-limb function. Several studies have quantitatively evaluated the amount of upper-limb activity, but few have measured finger usage. In this study, we used a ring-shaped wearable device to measure upper-limb and finger usage simultaneously in hospitalized patients with hemiplegic stroke and investigated the association between finger usage and general clinical evaluation.

**Methods:**

Twenty patients with hemiplegic stroke in an inpatient hospital participated in this study. All patients wore a ring-shaped wearable device on both hands for 9 h on the day of the intervention, and their finger and upper-limb usage were recorded. For the rehabilitation outcome assessments, the Fugl-Meyer Assessment of the Upper Extremity (FMA-UE), Simple Test for Evaluating Hand Function (STEF), Action Research Arm Test (ARAT), Motor Activity Log-14 (MAL), and Functional Independence Measure Motor (FIM-m) were performed and evaluated on the same day as the intervention.

**Results:**

Finger usage of the affected hand was moderately correlated with STEF ($r=0.48$, $p<0.05$) and STEF ratio ($r=0.47$, $p<0.05$). The finger-usage ratio was moderately correlated with FMA-UE ($r=0.56$, $p<0.05$) and ARAT ($r=0.53$, $p<0.05$), and strongly correlated with STEF ($r=0.80$, $p<0.01$) and STEF ratio ($r=0.80$, $p<0.01$). The upper-limb usage of the affected side was moderately correlated with FMA-UE ($r=0.46$, $p<0.05$), STEF ($r=0.55$, $p<0.05$) and STEF ratio ($r=0.54$, $p<0.05$), and strongly correlated with ARAT ($r=0.57$, $p<0.01$). The upper-limb usage ratio was moderately correlated with ARAT ($r=0.48$, $p<0.05$) and STEF ($r=0.55$, $p<0.05$), and strongly correlated with the STEF ratio ($r=0.61$, $p<0.01$). By contrast, there was no correlation between MAL and any of the measurements.

**Conclusions:**

This measurement technique provided useful information that was not biased by the subjectivity of the patients and therapists.

## Background

Stroke is one of the most common diseases worldwide [1]. Most stroke survivors experience some degree of upper limb dysfunction because of motor paralysis [2, 3]. Patients with stroke hemiplegia tend to reduce their frequency of using the affected upper limbs in activities of daily living and sometimes only use their unaffected limbs, resulting in a learned nonuse phenomenon [4, 5]. Learned nonuse is a major clinical problem impeding the recovery of motor function in affected limb and reducing the patient’s quality of life (QOL) [6]. Previous reports have stated that active use of the affected limb promotes use-dependent plasticity of the cerebral cortex [7-9], increasing the frequency of use of the affected limb in daily life is crucial for preventing learned nonuse and recovering from motor dysfunction. Another study reported that increased frequency of use of affected hands rather than improved function of affected hands contributed to patient QOL [10]. Therefore, encouraging the use of the affected limb in daily life by the therapist is important in rehabilitation.

The Motor Activity Log, developed by Taub et al, has been widely used as an evaluation method for assessing the amount of upper-limb activity in daily life [11]. This is a semi-structured interview for patients with hemiparetic stroke to assess the use of their paretic arm and hand from two aspects: the amount of use (AOU) and quality of movement (QOM). The Motor Activity Log-14 (MAL), which is generalized and commonly used, allows patients to self-evaluate AOU and QOM on a 6-point scale from 0 to 5 for 14 activities of daily living movement items [12, 13] and has been reported to have high reliability and excellent validity [14, 15]. Since MAL is an interview-style subjective evaluation scale, there is a potential problem is that the answer is significantly influenced by a patient’s cognitive level and subjectivity. Therefore, it is more effective to directly measure the amount of real-world arm use as an objective quantitative evaluation. 

Accelerometry is a method of recording the usage of the upper limbs using a watch-type wearable device with an embedded accelerometer [16, 17]. It is small, lightweight, and can implicitly measure the amount of affected limbs usage, so it is often used to measure upper-limb usage in daily life. Changes in upper-limb usage after rehabilitation intervention can be quantified, and the measurement data in clinics and research institutes have shown a high correlation with the assessments of upper-extremity function [18-20]. However, this method is unable to measure the finger usage during skilled movements because the accelerometer is attached to the wrist. Several devices, such as the Data Glove [21-23], and the Leap Motion [24, 25], have been recently developed for accurate finger-motion sensing and measuring of individual finger motions. Although these devices can perform accurate finger-motion measurements, they are not well-suited for continuous measurement of finger usage in patients with hemiplegic stroke because the daily usage had not been considered in their development. The Manumeter [26, 27] is a daily usable device using a magnetic field sensor and a ring-shaped magnet to measure the extent of wrist and finger usage. However, previous testing has shown that the correlation between the estimated and actual finger angles was not high ($R^2 \le 0.61$). The present study aim was to evaluate a newly-developed ring-shaped wearable device for a constant measurement of finger usage in daily life [28]. This device has many advantages, such as high measurement accuracy ($R^2 = 0.99$), easy attachment-detachment, and constant measurement. In addition, by recording the angular variation of the proximal inter-phalangeal (PIP) joint and defining the accumulative change as the amount of finger usage, quantitative measurements in daily life has been achieved.

To clarify the amount of finger usage in daily life in patients with hemiplegic stroke, quantitative measurements were made in the subacute phase of the hemiplegic stroke in patients who wore a ring-shaped wearable device. The amount of upper-limb usage was also measured simultaneously with an accelerometer incorporated into a wrist data recording unit to confirm consistency with the findings of previous studies. In addition, the correlations between daytime finger and upper-limb usage measured by the devices and respective clinical assessments were investigated to examine the hypothesis that patients with higher clinical evaluation scores should tend to use their fingers more frequently. This measurement technique is expected to provide additional universal evaluation results that are not biased by the subjectivity of patients and therapists.

## Methods

### Participants

Twenty patients with hemiplegic stroke in an inpatient hospital participated in this study. The inclusion criteria were (1) first-ever stroke (infarction or hemorrhage), (2) one-sided paralysis only, (3) no serious neurological or musculoskeletal problems prior to stroke, (4) no cognitive function problems (Mini-Mental State Examination > 23), (5) mild-to-moderate upper-limb paralysis assessed by the Fugl-Meyer Assessment of the Upper Extremity (FMA-UE > 19) [29]. The exclusion criteria were (1) unstable medical conditions and (2) patients restricted in their activities of daily living such as being instructed to stay on bed rest. The sample size was determined by considering the effect size (0.6), power (0.8), and significance level (0.05). According to the Declaration of Helsinki, all participants were informed of the study protocol, stress, possible risks, and their freedom to stop participating at any time, and gave their written informed consent. This study was conducted with the approval of the ethics committee of the Shonan Keiiku Hospital (19-002).

### Devices

**Fig. 1:**
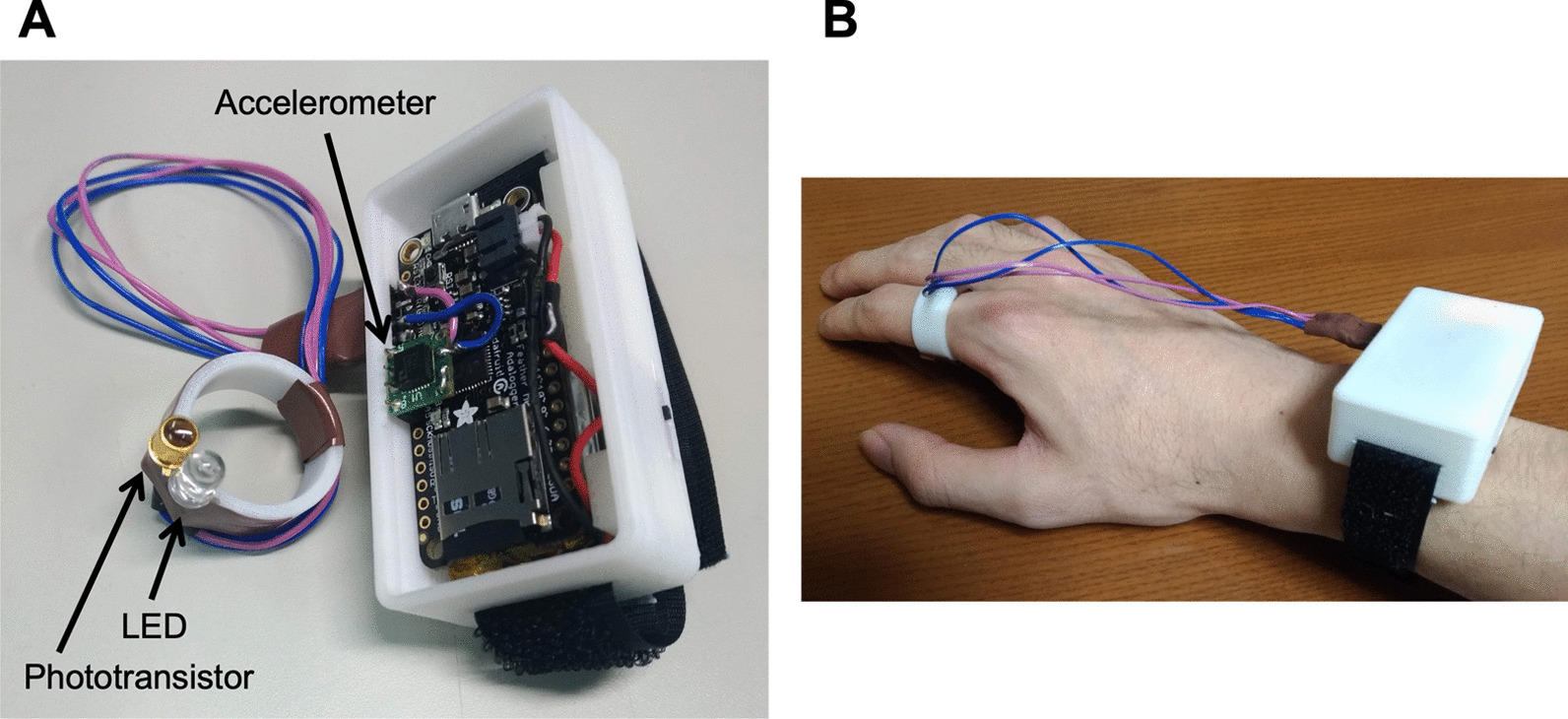
The developed ring device. **A** The ring device consists of an LED and a phototransistor. **B** The ring device was worn on the proximal phalanx of the index finger. A microcomputer, an accelerometer, and a battery were installed in a white wrist box

We developed a ring-shaped wearable device capable of measuring hand movements and estimating the flexion angle of each finger [28]. As shown in Fig. 1A, the device consists of a light-emitting diode (LED) (Osram, SFH4550) and a phototransistor (Honeywell, SD5410) [30]. The device was worn on the proximal phalanx of the index finger (Fig. 1B). The microcomputer (Adafruit, Feather M0 Adalogger) and a LiPo battery (3.7 V, 400 mAh) was installed in a white wrist box.

The ring-shaped device measures the distance between the fingertip and the device (*d*(*n*)) at 100 Hz. Based on a pre-measured length between the fingertip and PIP joint (*L*), the flexion angle of the PIP joint $\theta (n)$ can be estimated as $\theta (n) = \arccos ( d(n)/L )$, where *n* is the time-step sampled every 10 ms [28]. The cumulative angular change is recorded as finger usage, and the cumulative norm of the wrist accelerometer is simultaneously recorded as upper-limb usage [31, 32]. Simultaneous measurement with wrist and finger sensors enables separate finger movements from whole upper limb movements.

### Procedures

All patients wore the ring-shaped devices on both hands during the intervention period from 8:00 to 20:00, excluding the 3 h of rehabilitation intervention, for a total of 9 h. For the outcome assessments of the rehabilitation, the FMA-UE [33], Simple Test for Evaluating Hand Function (STEF) [34], Action Research Arm Test (ARAT) [35], MAL, and Functional Independence Measure Motor (FIM-m) [36] were performed and evaluated on the same day as the measurement. The reliability of each test has previously been confirmed. The MAL evaluation was performed by trained therapists (author NY). Other assessments (FMA-UE, STEF, ARAT and FIM-m) were performed in routine clinical practice by physical therapists, occupational therapists, and speech therapists under the supervision of author NY. Only general rehabilitation treatments were applied and no specific interventions were used in this study.

After completing the experiment, participants answered a post-survey questionnaire regarding the usability of the ring device. Participants were asked the following three questions using a 7-point Likert scale ranging from 0 to 7; (1) “Regarding ease of attaching,” (ranging from “difficult” to “easy”), (2) “There were restrictions on movement,” (ranging from “very restrictive” to “no restrictions”), and (3) “Regarding uncomfortableness when wearing,” (ranging from “very uncomfortable” to “no uncomfortable feeling”). The questionnaire also included open-ended responses.

### Statistical analysis

The Shapiro-Wilk test was used to evaluate the normality of the dataset distribution. Because all clinical measures were not normally distributed, a Wilcoxon signed-rank test was used to assess the difference in frequency of use between affected and unaffected hands. Finger usage, upper-limb usage, and all clinical measures were assessed by performing Spearman’s correlation test. The significance level was set at $p<0.05$. SPSS statistical software (Version 26, IBM SPSS Statistics for Windows, Armonk, NY) was used to perform all statistical analysis.

## Results

**Fig. 2:**
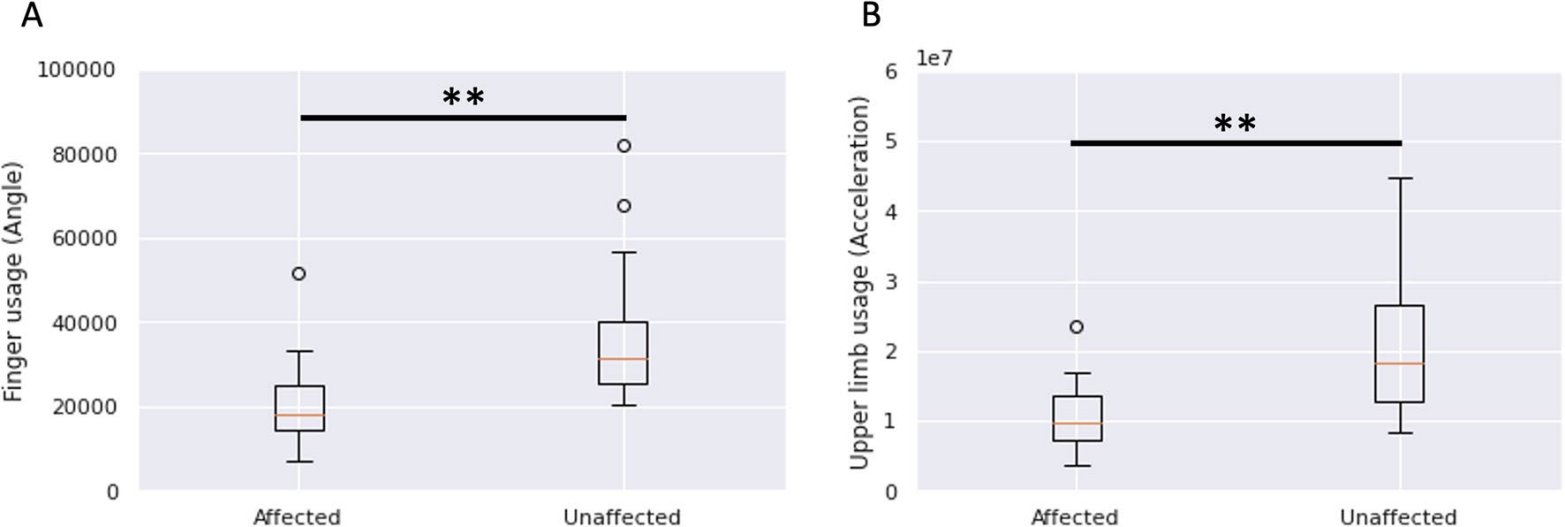
Comparison of finger usage (**A**) and upper-limb usage (**B**) between the affected side and unaffected side

Table 1 shows the characteristics of the participants and their respective scores for clinical assessments. Figure 2A shows the amount of the finger usage of the affected and unaffected hands per hour of the measured day (total of 9 h). The amount of finger usage was significantly smaller for the affected hand than for the unaffected hand ($p<0.01$). Figure 2B shows the amount of the upper-limb usage of the affected and unaffected hands per hour of the measured day. The amount of upper-limb usage was significantly smaller for the affected hand than for the unaffected hand ($p<0.01$).

**Table 1:** Participants’ characteristics

Variables	Values
Age	67.7 (13.6)
Sex (male/female)	11/9
Body mass index, kg/m^2^	22.6 (2.5)
Diagnosis (infarction/hemorrhage)	15/5
Dominant side (right/left)	20/0
Paresis side (right/left)	10/10
Days after the stroke	60.7 (32.3)
FMA-UE	52.1 (7.2)
STEF	
Affected side	60.6 (17.2)
Unaffected side	92.8 (7.0)
Affected/unaffected ratio	0.65 (0.19)
ARAT	
Affected side	47.0 (17.1)
Unaffected side	57 (0)
Affected/unaffected ratio	0.82 (0.14)
MAL	
Amount of use	2.7 (1.4)
Quality of movement	2.7 (1.2)
FIM	
Motor	80.1 (10.5)
Cognitive	34.2 (1.5)

The values are given as the mean (SD)

Table 2 shows the results of the correlation analysis between the finger usage, finger-usage ratio (affected hand/unaffected hand), and each clinical assessment measure. The finger usage of the affected hand was moderately correlated with the STEF ($r=0.48$, $p<0.05$) and STEF ratio ($r=0.47$, $p<0.05$). The finger-usage ratio was moderately correlated with the FMA-UE ($r=0.56$, $p<0.05$) and ARAT ($r=0.53$, $p<0.05$) and strongly correlated with the STEF ($r=0.80$, $p<0.01$) and STEF ratio ($r=0.80$, $p<0.01$). On the other hand, neither the finger usage on the affected hand nor the finger-usage ratio were significantly correlated with MAL AOU (finger usage: $p=0.41$, finger-usage ratio: $p=0.27$) and MAL QOM (finger usage: $p=0.42$, finger-usage ratio: $p=0.29$).

**Table 2:** Correlation coefficients (Finger)

Clinical measurements	Finger usage (affected side)	Finger-usage ratio (affected/unaffected side)
FMA-UE	0.41	0.56*
ARAT		
Affected side	0.44	0.53*
STEF		
Affected side	0.48*	0.80**
Unaffected side	0.12	0.01
Ratio (affected/unaffected)	0.47*	0.80**
MAL		
Amount of use	0.20	0.26
Quality of movement	0.19	0.25
FIM-m	0.43	0.25

*$$p<0.05$$, **$$p<0.01$$

Table 3 shows the results of the correlation analysis between the upper-limb usage, upper-limb usage ratio (affected hand/unaffected hand), and each clinical assessment measure. The upper-limb usage of the affected side was moderately correlated with the FMA-UE ($r=0.46$, $p<0.05$), STEF ($r=0.55$, $p<0.05$), and STEF ratio ($r=0.54$, $p<0.05$) and strongly correlated with ARAT ($r=0.57$, $p<0.01$). The upper-limb-usage ratio was moderately correlated with ARAT ($r=0.48$, $p<0.05$) and STEF ($r=0.55$, $p<0.05$), and strongly correlated with the STEF ratio ($r=0.61$, $p<0.01$). On the other hand, neither the upper-limb usage on the affected side nor the upper-limb-usage ratio were significantly correlated with MAL AOU (upper-limb usage: $p=0.30$, upper-limb-usage ratio: $p=0.66$) and MAL QOM (upper-limb usage: $p=0.26$, upper-limb-usage ratio: $p=0.67$).

**Table 3:** Correlation coefficients (Upper-limb)

Clinical measurements	Upper-limb usage (affected side)	Upper-limb-usage ratio (affected/unaffected side)
FMA-UE	0.46*	0.37
ARAT		
Affected side	0.57**	0.48*
STEF		
Affected side	0.55*	0.55*
Unaffected side	0.19	0.21
Ratio (affected/unaffected)	0.54*	0.61**
MAL		
Amount of use	0.24	0.10
Quality of movement	0.26	0.10
FIM-m	0.36	0.01

*$$p<0.05$$, **$$p<0.01$$

**Fig. 3:**
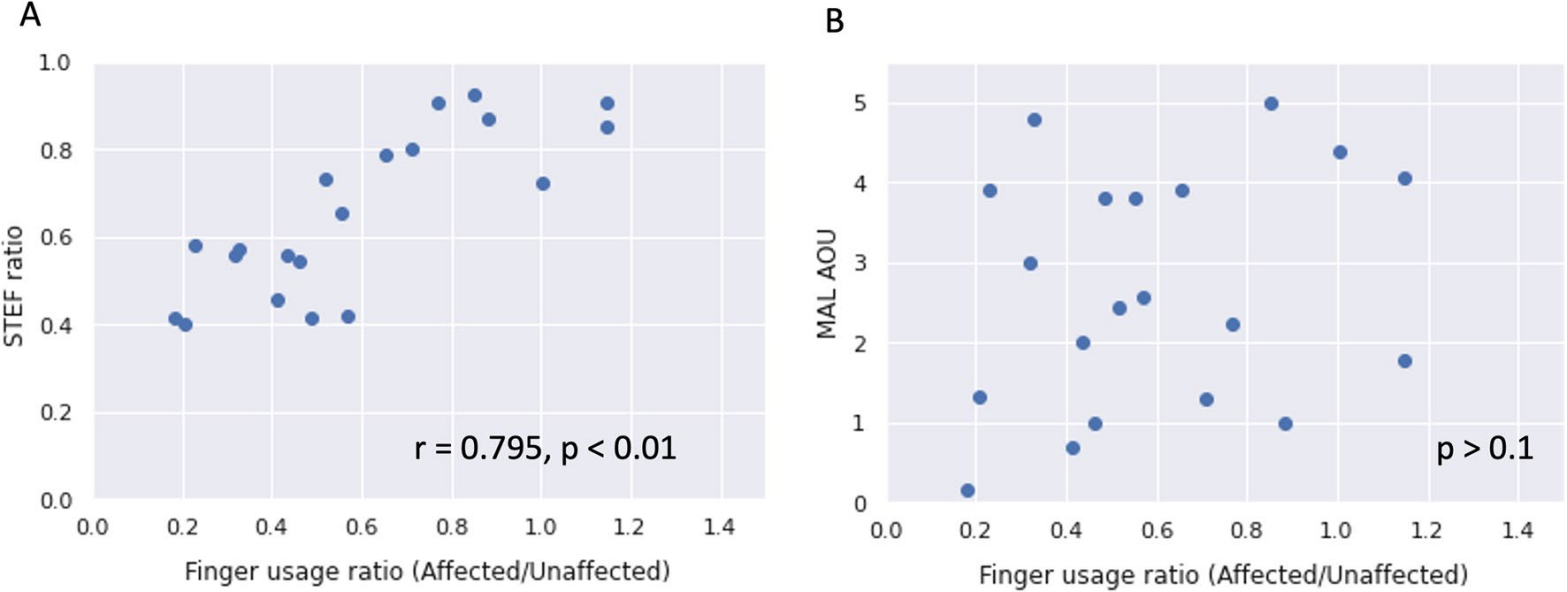
Correlation analysis of the finger-usage ratio and STEF/MAL(AOU)

**Fig. 4:**
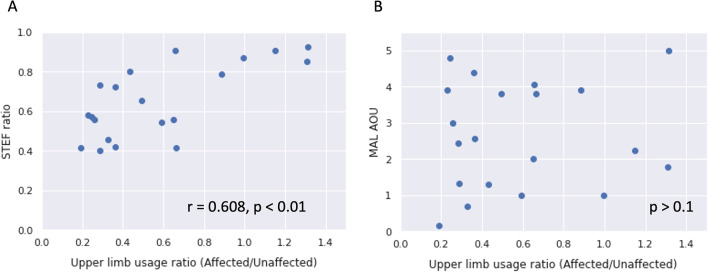
Correlation analysis of upper-limb usage ratio and STEF/MAL(AOU)

Figure 3A shows the relationship between the finger-usage ratio and STEF ratio as a representative example of a strong correlation with the finger-usage ratio. The relationship with MAL (AOU) is shown in Fig. 3B as a representative example of no correlation. Figure 4A shows the relationship between the upper-limb-usage ratio and STEF ratio as a representative example of a strong correlation with upper-limb-usage ratio. The relationship with MAL (AOU) is shown in Fig. 4B as a representative example of no correlation.

The mean scores (and SD) for the three questions about wearing comfort were (1) 6.70 (0.46) for “Regarding ease of attaching,” (2) 6.55 (0.59) for “There were restrictions on movement,” and (3) 6.40 (0.67) for “Regarding uncomfortableness when wearing.” Comments from the free response section of the questionnaire included, such as, “There were many sizes of rings available, so I was able to use the right size,” and “The device is lightweight, so I can spend my daily life without any discomfort.”

## Discussion

In most previous studies, the amount of real-world arm use was estimated by wearing an accelerometer on the wrist. In the present study, a newly-developed ring-shaped wearable device was used to measure the skilled motor activities of the fingers separate from those of the wrist. Investigation of the relationship between the quantitative measurements made with the device and each clinical measurement scores provided the following information.

First, both the finger usage and upper-limb usage were significantly smaller on the affected side than on he unaffected side. In the previous study by Lang et al. [18], an accelerometer was used to measure real-world arm usage, and the ratio of the affected-hand usage to the unaffected-hand usage was approximately 55%. In this study, the amount of finger usage for the affected hand was approximately 59% of that for the unaffected hand, and the amount of upper-limb usage on the affected side was approximately 58% of that on the unaffected side, so the results were similar to those of Lang et al.

Secondly, the finger-usage ratio measured in this study correlated with the FMA-UE, ARAT, and STEF, and the upper-limb-usage ratio also correlated with ARAT and STEF. The correlation between the clinical measurements of upper-limb function and finger usage in daily life was consistent with our hypothesis. Previous studies that used accelerometers to measure the amount of real-world arm use showed a correlation with FMA-UE [37] and ARAT [18], which was almost consistent with this study. Regarding STEF, no previous study has quantitatively compared the activities in daily life. Thus, this study is the first to investigate the correlation between finger usage and STEF. Among the clinical measures used in this study, STEF showed the highest correlation with finger usage. Since STEF is mainly used to evaluate finger function in the upper limbs, this correlation was considered to be particularly relevant. FMA-UE, ARAT, and STEF are specific measurements indicating the function of the upper limbs. Overall, the finding that these measures correlated with finger usage supports the construct validity of this study.

Third, this is the most notable result of this study, and contrary to our hypothesis, no correlation was found between the finger and upper-limb usage and MAL scores. This means that there was a dissociation between MAL, which is a subjective evaluation of the real-world arm use, and the quantitative evaluation of the finger and upper limb usage measured in this study. MAL is the main standard clinical evaluation for assessing the amount and quality of use of affected limbs in daily life. Previous studies measuring upper extremity usage with an accelerometer have found a moderate correlation between the real-world arm use and MAL [13, 38-40]. Therefore, it was also expected that there would be a correlation between MAL and the finger usage measured in this study, but no correlation was observed. Two important points warrant further discussion. First, all subjects in this study were inpatients. In most previous studies, MAL was evaluated in community-dwelling patients with stroke under mild conditions [13, 38, 39]. The MAL is a clinical measure of the amount and quality of use for 14 items in daily life. During hospitalization, much of daily life is restricted, and the range of activities is narrowed. Therefore, some actions not performed during hospitalization were included among the 14 items, which is considered to have affected the MAL score. One previous study measured the amount of activity in hospitalized stroke patients with an accelerometer [40], but that study targeted patients in the acute phase shortly after onset, and the measurement timing was significantly different from that of the present study. These differences in patient characteristics are thought to have caused the discrepancy in the results. Another point is that MAL is an interview-style evaluation. Patients with stroke occasionally have some deficits, such as memory problems, aphasia, and dementia. Since MAL is an evaluation asking such patients subjectively how much their own hands have been used [41, 42], it is necessary to determine whether the patients properly evaluate their real-world arm use in daily life. Additionally, when assessing MAL, it is necessary to be aware of the Hawthorne effect [43], which is caused by the patient feeling that therapists are expecting a certain outcome of the intervention. The irrelevancy of the finger usage measured objectively in this study suggests evaluations should note whether or not a patient was able to perform an appropriate self-evaluation in MAL. The problem of MAL as an evaluation tool was also highlighted.

No correlation was revealed between either the finger and upper-limb usage and FIM-m. The results in this study indicate that higher self-care independence does not mean that the affected limb use is more frequently used. The FIM-m evaluation reflects the amount of assistance required in daily life, but the evaluation target is the entire motor activity including on the affected and unaffected sides. Therefore, the evaluation characteristically shows a high score even in patients who have independent compensatory movement without using the affected limb. A previous study by Lang et al. [18] found a correlation between FIM and upper-extremity use measured using an accelerometer. This previous study targeted patients in the acute phase, a period focused on restoring function through the positive use of the affected limb, and it is thought that upper-limb usage of the affected side influenced the independence. On the other hand, the patients in the subacute phase who participated in this study were in a rehabilitation program with an emphasis on independence, and there were no specific recommendations to use the affected limb or limit compensatory movements of the unaffected limb in this measurement. The results of a questionnaire conducted after the measurements revealed that usability of the ring device was good and there were no restrictions on daily activities due to wearing the device.

All clinical measures used in this study were assessed according to the therapist’s and patient’s subjective evaluation. Therefore, future clinical measurements should be performed by an experienced and skilled evaluator. Murphy et al. stated that objective evaluations, such as accelerometers, helped close the gap between device-based evaluations and traditional clinical evaluation outcomes and improve the quality and accuracy of measurements [44]. The objective evaluation scores measured using this new device suggest that the variability within and between patients could be reduced relative to the variability of the clinical evaluation scores. In addition, it might be possible to provide useful information to patients and therapists by adding objective evaluations, such as finger usage in daily life, to the conventional subjective evaluations.

This study had several limitations. First, the study participants were recruited at only a single institution. Therefore, compared with community-dwelling patients, the participants’ activities of daily living were restricted, compared to community-dwelling patients, and their group daily life schedules might have become more similar with less individuality. Future investigations are needed to determine if the same trend extends to other types of institutions. Second, the subjects were not randomly selected but were recruited from patients willing to participate in a clinical trial. Patients who had no voluntary upper-extremity movements or who had recovered near-normal levels of upper-extremity function were excluded. Therefore, all patients in this trial had mild to moderate hemiplegia, with a narrow range of severity. Although such patients are in an appropriate target range because they have symptoms relevant to many studies of upper-extremity rehabilitation, more studies are required to expand the target to patients with more diverse severity in the future. Third, in this study, only the movement of a single finger was measured. However, it is possible to attach this device to more than one finger to measure a variety of hand motions in the future. Fourth, although the results of the usability questionnaire were relatively good, this study only included inpatients, so it is not possible to determine the usability in daily life in the community after discharge. To adapt this device to patients living in the community in the future, it is necessary to conduct usability evaluation by brushing up the content of the questions.

The ring device can measure the usage of the upper-limb and fingers simultaneously and individually. Focusing on the use of fingers as well as upper-limbs would be significant for a deeper understanding of more skilled movements such as folding a towel, buttoning, or picking up a meal and bringing it to the mouth.

## Conclusions

This study performed quantitative evaluations of the upper-limb and finger usage in the daily lives of patients with hemiplegic stroke, and our results showed that the newly developed ring-shaped wearable devices could measure not only the amount of real-world arm usage but also the amount of finger usage, such as the skilled motor activities of fingers separately from the activities of the wrist. These results provide an objective evaluation index for finger usage, which could conventionally only be obtained from subjective self-reported evaluations. This measurement technique is expected to provide universal information without the subjective biases of patients and therapists.

## Data Availability

The datasets used and/or analyzed during this study are available from the corresponding author upon reasonable request.

## Abbreviations

FMA-UE: Fugl-Meyer Assessment of the Upper Extremity
STEF: Simple Test for Evaluating Hand Function
ARAT: Action Research Arm Test
MAL: Motor Activity Log-14
AOU: Amount of use
QOM: Quality of movement
FIM-m: Functional Independence Measure Motor
QOL: Quality of life
LED: Light emitting diode
PIP: Proximal inter-phalangeal

## References

[1] Ward NS, Cohen LG (2004). Mechanisms underlying recovery of motor function after stroke. Arch Neurol.

[2] Broeks JG, Lankhorst GJ, Rumping K, Prevo AJ (1999). The long-term outcome of arm function after stroke: results of a follow-up study. Disabil Rehabil.

[3] Wolf SL, Winstein CJ, Miller JP, Taub E, Uswatte G, Morris D, Giuliani C, Light KE, Nichols-Larsen D (2006). Effect of constraint-induced movement therapy on upper extremity function 3 to 9 months after stroke: the EXCITE randomized clinical trial. Disabil Rehabil.

[4] Taub E, Uswatte G, Elbert T (2002). New treatments in neurorehabilitation founded on basic research. Nat Rev Neurosci.

[5] Taub E, Uswatte G, Mark VW, Morris DM (2006). The learned nonuse phenomenon: implications for rehabilitation. Eura Medicophys.

[6] Nichols-Larsen DS, Clark PC, Zeringue A, Greenspan A, Blanton S (2005). Factors influencing stroke survivors’ quality of life during subacute recovery. Stroke.

[7] Grefkes C, Nowak DA, Eickhoff SB, Dafotakis M, Küst J, Karbe H, Fink GR (2008). Cortical connectivity after subcortical stroke assessed with functional magnetic resonance imaging. Ann Neurol.

[8] Walther M, Juenger H, Kuhnke N, Wilke M, Brodbeck V, Berweck S, Staudt M, Mall V (2009). Motor cortex plasticity in ischemic perinatal stroke: a transcranial magnetic stimulation and functional MRI study. Pediatr Neurol.

[9] Liepert J, Bauder H, Wolfgang HR, Miltner WH, Taub E, Weiller C (2000). Treatment-induced cortical reorganization after stroke in humans. Stroke.

[10] Kelly KM, Borstad AL, Kline D, Gauthier LV (2018). Improved quality of life following constraint-induced movement therapy is associated with gains in arm use, but not motor improvement. Top Stroke Rehabil.

[11] Taub E, Miller NE, Novack TA, Cook EW, Fleming WC, Nepomuceno CS, Connell JS, Crago JE (1993). Technique to improve chronic motor deficit after stroke. Arch Phys Med Rehabil.

[12] van der Lee JH, Beckerman H, Knol DL, de Vet HC, Bouter LM (2004). Clinimetric properties of the motor activity log for the assessment of arm use in hemiparetic patients. Stroke.

[13] Uswatte G, Taub E, Morris D, Vignolo M, McCulloch K (2005). Reliability and validity of the upper-extremity Motor Activity Log-14 for measuring real-world arm use. Stroke.

[14] Pereira ND, Ovando AC, Michaelsen SM, Anjos SM, Lima RC, Nascimento LR, Teixeira-Salmela LF (2012). Motor Activity Log-Brazil: reliability and relationships with motor impairments in individuals with chronic stroke. Arquivos de neuro-psiquiatria.

[15] Lin KC, Chuang LL, Wu CY, Hsieh YW, Chang WY (2010). Responsiveness and validity of three dexterous function measures in stroke rehabilitation. J Rehabil Res Dev.

[16] Noorkoiv M, Rodgers H, Price CI (2014). Accelerometer measurement of upper extremity movement after stroke: a systematic review of clinical studies. J Neuroeng Rehabil.

[17] Gebruers N, Vanroy C, Truijen S, Engelborghs S, De Deyn PP (2010). Monitoring of physical activity after stroke: a systematic review of accelerometry-based measures. Arch Phys Med Rehabil.

[18] Lang CE, Wagner JM, Edwards DF, Dromerick AW (2007). Upper extremity use in people with hemiparesis in the first few weeks after stroke. J Neurol Phys Ther.

[19] Uswatte G, Miltner WH, Foo B, Varma M, Moran S, Taub E (2000). Objective measurement of functional upper-extremity movement using accelerometer recordings transformed with a threshold filter. Stroke.

[20] Uswatte G, Giuliani C, Winstein C, Zeringue A, Hobbs L, Wolf SL (2006). Validity of accelerometry for monitoring real-world arm activity in patients with subacute stroke: evidence from the extremity constraint-induced therapy evaluation trial. Arch Phys Med Rehabil.

[21] Lin BS, Lee IJ, Yang SY, Lo YC, Lee J, Chen JL (2018). Design of an inertial-sensor-based data glove for hand function evaluation. Sensors.

[22] Tarchanidis KN, Lygouras JN (2003). Data glove with a force sensor. IEEE Trans Instrum Meas.

[23] Simone LK, Kamper DG (2005). Design considerations for a wearable monitor to measure finger posture. J Neuroeng Rehabil.

[24] Ultraleap Limited. Leap motion. https://www.ultraleap.com/. Accessed 18 Feb 2022.

[25] Colombini G, Duradoni M, Carpi F, Vagnoli L, Guazzini A (2021). LEAP motion technology and psychology: a mini-review on hand movements sensing for neurodevelopmental and neurocognitive disorders. Int J Environ Res Public Health.

[26] Rowe JB, Friedman N, Bachman M, Reinkensmeyer DJ (2013). The manumeter: a non-obtrusive wearable device for monitoring spontaneous use of the wrist and fingers. IEEE Int Conf Rehabil Robot.

[27] Friedman N, Rowe JB, Reinkensmeyer DJ, Bachman M (2014). The manumeter: a wearable device for monitoring daily use of the wrist and fingers. IEEE J Biomed Health Inform.

[28] Yamamoto N, Matsumoto T, Sudo T, Megumi M, Kondo T. Ring-shaped wearable device for logging finger usage in daily life. In: 2022 international symposium on micro-nanomechatronics and human science (MHS); 2022. p. 1–6. 10.1109/MHS56725.2022.10092178.

[29] Woodbury ML, Velozo CA, Richards LG, Duncan PW (2013). Rasch analysis staging methodology to classify upper extremity movement impairment after stroke. Arch Phys Med Rehabil.

[30] Kienzle W, Hinckley K. Lightring: Always-available 2d input on any surface. In: UIST ’14 proceedings of the 27th annual ACM symposium on user interface software and technology. 2014; p. 157–160.

[31] Bezuidenhout L, Joseph C, Einarsson U, Thurston C, Maria H, Moulaee Conradsson D (2022). Accelerometer assessed upper limb activity in people with stroke: a validation study considering ambulatory and non-ambulatory activities. Disabil Rehabil.

[32] Leeger-Aschmann CS, Schmutz EA, Zysset AE, Kakebeeke TH, Messerli-Bürgy N, Stülb K, Arhab A, Meyer AH, Munsch S, Jenni OG, Puder JJ, Kriemler S (2019). Accelerometer-derived physical activity estimation in preschoolers—comparison of cut-point sets incorporating the vector magnitude vs the vertical axis. BMC Public Health.

[33] Fugl-Meyer AR, Jääskö L, Leyman I, Olsson S, Steglind S (1975). The post-stroke hemiplegic patient. 1. A method for evaluation of physical performance. Scand J Rehabil Med.

[34] Shindo K, Oba H, Hara J, Ito M, Hotta F, Liu M (2015). Psychometric properties of the simple test for evaluating hand function in patients with stroke. Brain Injury.

[35] Lyle RC (1981). A performance test for assessment of upper limb function in physical rehabilitation treatment and research. Int J Rehabil Res.

[36] Granger CV, Cotter AC, Hamilton BB, Fiedler RC (1993). Functional assessment scales: a study of persons after stroke. Arch Phys Med Rehabil.

[37] Gebruers N, Truijen S, Engelborghs S, Nagels G, Brouns R, De Deyn PP (2008). Actigraphic measurement of motor deficits in acute ischemic stroke. Cerebrovasc Dis.

[38] Uswatte G, Taub E, Morris D, Light K, Thompson PA (2006). The Motor Activity Log-28: assessing daily use of the hemiparetic arm after stroke. Neurology.

[39] Uswatte G, Foo WL, Olmstead H, Lopez K, Holand A, Simms LB (2005). Ambulatory monitoring of arm movement using accelerometry: an objective measure of upper-extremity rehabilitation in persons with chronic stroke. Arch Phys Med Rehabil.

[40] Narai E, Hagino H, Komatsu T, Togo F (2016). Accelerometer-based monitoring of upper limb movement in older adults with acute and subacute stroke. J Geriatr Phys Ther.

[41] Bradburn NM, Rips LJ, Shevell SK (1987). Answering autobiographical questions: the impact of memory and inference on surveys. Science.

[42] Tatemichi TK, Desmond DW, Stern Y, Paik M, Sano M, Bagiella E (1994). Cognitive impairment after stroke: frequency, patterns, and relationship to functional abilities. J Neurol Neurosurg Psychiatry.

[43] Wolf SL, Blanton S, Baer H, Breshears J, Butler AJ (2002). Repetitive task practice: a critical review of constraint-induced movement therapy in stroke. Neurologist.

[44] Alt Murphy M, Resteghini C, Feys P, Lamers I (2015). An overview of systematic reviews on upper extremity outcome measures after stroke. BMC Neurol.

